# Tocilizumab reduces COVID-19 mortality and pathology in a dose and timing-dependent fashion: a multi-centric study

**DOI:** 10.1038/s41598-021-99291-z

**Published:** 2021-10-05

**Authors:** Alejandro Durán-Méndez, Alma Delia Aguilar-Arroyo, Emiliano Vivanco-Gómez, Eduardo Nieto-Ortega, Daniela Pérez-Ortega, Cristian Jiménez-Pérez, Karla Y. Hernández-Skewes, Guillermo Montiel-Bravo, Oscar J. Roque-Reyes, Fernanda Romero-Lechuga, Diana Medina-Santos, Perla Oriana-Román, Jorge Rafael Flores-Hernández, Juan Daniel Méndez-Coca, Daniela Montaño-Olmos, Karla Cecilia Farfán-Lazos, Miranda Tobón-Cubillos, América Viveros-Hernández, Fernando Sevilla-Castillo, Ángel Raúl Hernández-Romero, Shannat Ortega-Rodríguez, Aldo Christiaan Jardínez-Vera, María Antonieta Solís-González, Antonio Ramos de la Medina, Laura Martínez Pérez-Maldonado, Elizabeth Lagunes-Lara, Miguel Cova-Bonilla, Alberto N. Peón

**Affiliations:** 1 Laboratorio Santiago Ramón y Cajal, Sociedad Española de Beneficencia, Av. Juárez #908, Col. La Villita, CP42060 Pachuca, Hidalgo Mexico; 2 Área Académica de Medicina, Universidad Autónoma del Estado de Hidalgo, Pachuca, Mexico; 3 Facultad de Medicina, Universidad Nacional Autónoma de México, Mexico, Mexico; 4 Facultad de Medicina, Benemérita Universidad Autónoma de Puebla, Mexico, Mexico; 5 Universidad Anáhuac, Puebla, Mexico, Mexico; 6 Escuela Superior de Apan, Universidad Autónoma del Estado de Hidalgo, Carretera Apan-Calpulalpan s/n, Colonia, 43920 Chimalpa Tlalayote, Hgo Mexico; 7 Centro de Investigación en Cirugía Global, Hospital Español, Veracruz, Mexico

**Keywords:** Viral infection, Molecularly targeted therapy, Outcomes research

## Abstract

Life-threatening COVID-19 is associated with strong inflammation, where an IL-6-driven cytokine storm appears to be a cornerstone for enhanced pathology. Nonetheless, the specific inhibition of such pathway has shown mixed outcomes. This could be due to variations in the dose of tocilizumab used, the stage in which the drug is administered or the severity of disease presentation. Thus, we performed a retrospective multicentric study in 140 patients with moderate to critical COVID-19, 79 of which received tocilizumab in variable standard doses (< 400 mg, 400–800 mg or > 800 mg), either at the viral (1–7 days post-symptom onset), early inflammatory (8–15) or late inflammatory (16 or more) stages, and compared it with standard treated patients. Mortality, reduced respiratory support requirements and pathology markers were measured. Tocilizumab significantly reduced the respiratory support requirements (OR 2.71, CI 1.37–4.85 at 95%) and inflammatory markers (OR 4.82, CI 1.4–15.8) of all patients, but mortality was only reduced (4.1% vs 25.7%, *p* = 0.03) when the drug was administered at the early inflammatory stage and in doses ranging 400–800 mg in severely-ill patients. Despite the apparent inability of Tocilizumab to prevent the progression of COVID-19 into a critical presentation, severely-ill patients may be benefited by its use in the early inflammatory stage and moderate doses.

## Introduction

The coronavirus disease-19 (COVID-19) is induced by the infection of the respiratory tract with the SARS-CoV-2 virus, which is a cytopathic agent that causes lung tissue damage by inducing pyroptosis^1,2^, which is a cytolytic and thus highly inflammatory kind of programmed cell death that usually occurs after intracellular infections^3^, and thus renders COVID-19 as not only a viral proliferation-dependent, but also an inflammation-driven illness.

Pioneering studies^4^ showed that critically-ill patients presented higher levels of inflammatory markers than patients with low to moderate illness, while dead patients had higher degrees of lymphopenia, neutrophilia, C-reactive protein (CRP), lactate dehydrogenase (LDH), d-dimer and IL-6 production^5^; all of these markers corresponding to a strong T-helper type 1 (Th1) immune response.

In consequence, a stereotypical natural history of COVID-19 has been proposed in which the first ≈ 7 days after symptom onset are dominated by viral shedding, with low inflammatory conditions^6^, thus being recognized as a viral stage (VS). At day ≈ 8 begins a stage where the viral shedding becomes mostly reduced whereas inflammation becomes more dominant, and patients tend to develop a severe form of the disease, thus being recognized as the early inflammatory stage (EIS). And if inflammation progresses beyond 16 days to a late inflammatory stage (LIS), critical disease with acute respiratory distress syndrome, acute cardiac injury, multi-organ failure and death are most likely to occur^7,8^.

Importantly, this line of thought leads to the hypothesis that the VS of the disease may be optimally treated with anti-viral agents, and without strong pleiotropic anti-inflammatory drugs^6^; whereas the inflammatory stage may be best controlled by the means of anti-inflammatory agents^9,10^, with little to no benefit from concomitant administration of anti-viral agents^11^.

Due to the fact that IL-6 is highly expressed in the severe to critical presentations of COVID-19^5^, many research teams have used Tocilizumab (Tcz), a humanized monoclonal antibody that targets the IL-6 receptor (IL-6R) in its membrane-bound and soluble forms^12^, to down-modulate inflammation, and thus to avoid respiratory and multi-organ dysfunction. These studies have shown varied results that range from a lack of effect on mortality, time to clinical improvement, laboratory parameters and/or prevention of progression to a more severe disease^13–16^, to success in the modulation of some or all of these parameters in moderately^17^, severely^18–22^, and/or critically-ill patients^23–25^.

Although such variations seem mostly promising, they pinpoint to the fact that no standardized Tcz dose or timing for administration into the different disease’s stages have been established, and that we mostly lack knowledge about the kind of patients that may be most benefited by the use of this drug. Another interpretation may be that other factors, such as different strains and patient’s characteristics, may play important roles in the pathophysiology of the disease^26^, or even that IL-6 importance into the whole phenomenon may be overestimated^27^, as its levels are not as high as in other pathologies where IL-6R-neutralization has shown consistent benefits. However, the standardization of dose and timing for administration may still represent a valuable step to ponder the role of IL-6 signaling into the disease’s pathophysiology, and thus in tocilizumab’s efficacy for the treatment of COVID-19.

Following this line of thought we performed a retrospective, observational, case controlled study on the use of Tcz in patients with moderate to critical COVID-19 presentations, studying three different standard dose intervals (< 400 mg, 400–800 mg and > 800 mg) and their administration on three different stages of the disease: VS, EIS and LIS.

## Materials and methods

Clinical files belonging to 260 COVID-19 positive patients in Hospital Español de Pachuca, Sociedad Española de Beneficencia (Pachuca) and Hospital Español de Veracruz hospitals from May 1st 2020 to July 1st 2021 were identified. The records were screened for the following inclusion criteria: (i) patients with positive test results for SARS-CoV-2, (ii) hospitalized patients after a moderate to severe COVID-19, (iii) files that showed laboratory evaluations in at least 80% of the in-hospital days, (iv) patients receiving any kind of respiratory support, and (v) patients with signed informed consent for the study. Then the exclusion criteria were used to eliminate files from: (i) voluntarily-discharged patients, (ii) patients that died 48 h upon admission (because the treatments may not had the optimal time to exert their effects), (iii) died at the same day of Tcz administration (because the treatment needs three days to achieve its maximum concentration), and (iv) patients with clinical files lacking crucial information.

Relevant data was collected on a Google Docs file, identifying two main treatment-groups: the standard treated (ST) (enoxaparin plus 6 mg of dexamethasone once daily) and the Tcz (ST plus varying Tcz standard doses). Data gathered was double checked by a blind researcher to ensure precision.

The severity of the disease in each patient was evaluated by the CALL score at hospital admission using an online calculator^28^. Patients were classified into three categories: (i) moderately-ill patients were those that required non-invasive respiratory support but accumulated up to seven points in the CALL score, (ii) severely-ill patients were those that required non-invasive respiratory support but had 8 or more points in the CALL score, and (iii) critically-ill patients were those that required mechanical ventilation. In either category there were patients that were treated with Tcz or ST.

Tcz was administered at an average of 3.5 days post-hospital admission, but as patients sought medical care at different disease stages, the treatments were performed at different time-points. When Tcz was administered at days 1–7 following symptom-development, the patients were considered as part of the VS group; but when the drug was used at 8–15 days, or 16 or more days after symptom-development, the patients were considered as a part of the EIS or LIS groups, respectively. Furthermore, patients were classified according to three dose intervals administered: < 400 mg, 400–800 mg and > 800 mg. Second doses were used when patients did not present a reduction of their CRP levels within 48 h, but patients were classified in either dose-group taking into account the total quantity of Tcz that they were administered.

The effect of Tcz on survival was evaluated by calculating the mortality of both groups (mortality % = (deaths × 100)/n). Differences in such data were evaluated by performing a Fisher’s exact test and considered significant when *p* values ≤ 0.05. The hospitalization length was quantified, averaged and compared by using a Student’s t test. All data were plotted into bar graphics using the GraphPad Prism 9 software. *p* values ≤ 0.05 were considered significant.

The odds ratios (OR) for the reduction of respiratory support requirements (RSR), the prevention of progression to mechanical ventilation (MV), the return to normal levels of leukocyte, lymphocyte, neutrophil, LDH, CRP, d-dimer and ferritin, all five days after the treatment with Tcz were quantified by using the online calculator VassarStats^29^. This time frame was chosen as this drug achieves its maximum concentration in three days and its maximum clinically observable effects at day 5 post-administration^30^.

A protocol for this study was evaluated by the Institutional Committee of Research Ethics of the Sociedad Española de Beneficencia (Pachuca, Hidalgo) and the study was approved on April 23th of 2020. Our sponsor had no role in study design. All methods were performed in accordance with the relevant guidelines and regulations, including the Declaration of Helsinki.

## Results

### Selection of clinical records and patient’s characteristics

**Figure 1:**
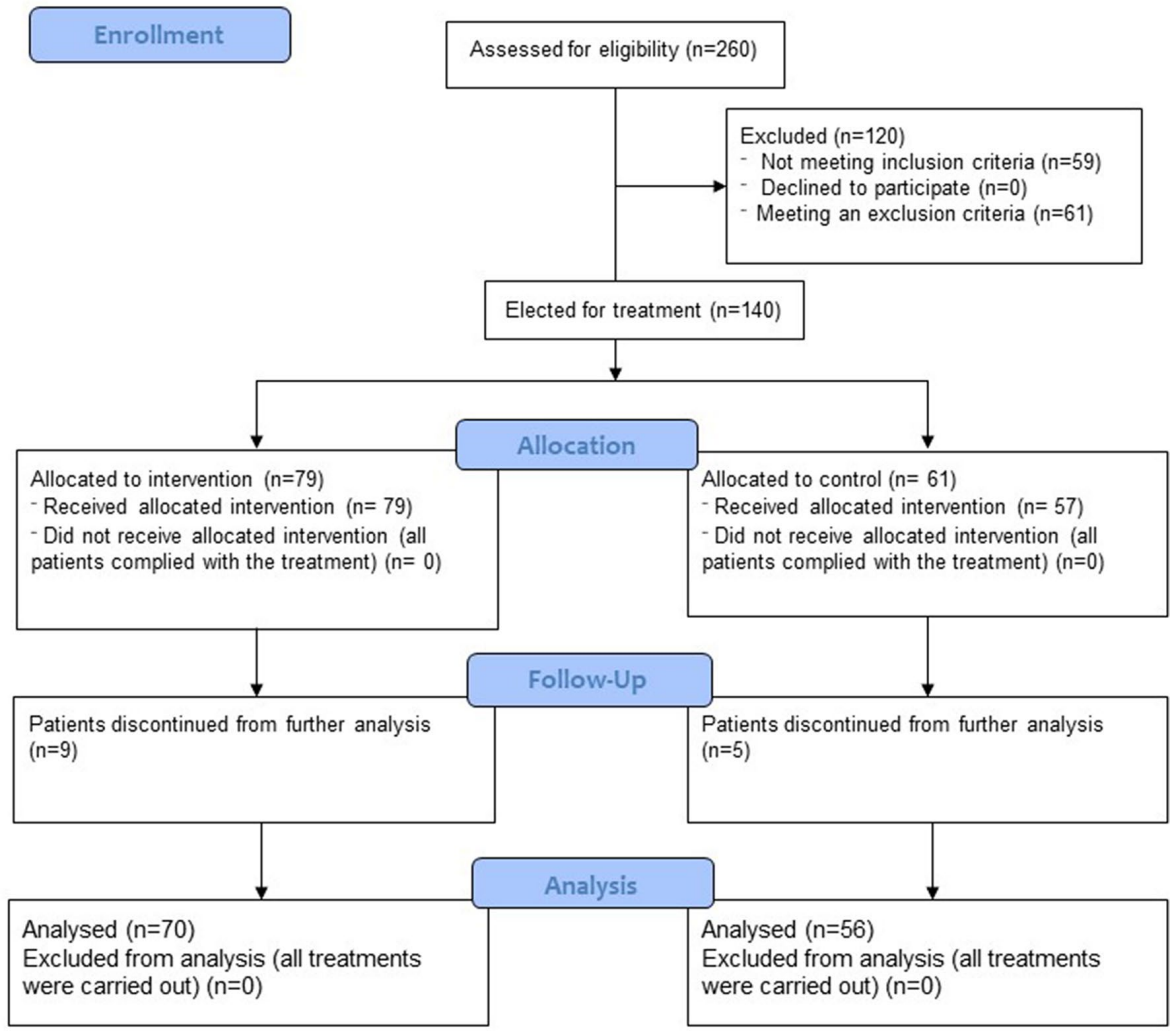
Screening and eligibility of clinical records. A total of 260 clinical files of SARS-CoV-2-positive patients were identified in three health centers in México. 201 met the inclusion criteria, while 61 met one or more exclusion criteria. In this way, 140 clinical files were included in the study, but 14 records were discontinued from further analysis as they belonged to critically-ill patients, and together did not render a statistically manageable sample.

After screening the databases of the aforementioned Hospitals in search for eligible clinical files, we identified 260 records. 201 matched our inclusion criteria, but only 140 of such could be included in the study after eliminating those that met any or all the exclusion criteria (Fig. 1). The files were sorted into two categories: the ST-patients (n = 61) and the Tcz-patients (n = 79). From the ST group 5 were critically-ill, 36 were severely-ill and 20 moderately-ill; accordingly, in the Tcz group 9 patients had a critical condition, 39 were severely-ill and 31 were moderately-ill. Moderately, severely and critically-ill patients were treated with either the ST or the Tcz regimes in variable doses and disease stages.

As shown in Supplementary Table S1 online Tcz treatments were administered at an average of 3.5 days post-hospital admission, and the average standard doses were 790 mg, 790 mg or 550 mg for the critically, severely and moderately-ill patients, respectively. While the majority (55.5%) of critically-ill patients received doses of > 800 mg, larger proportions of severely (25.6%) and moderately-ill (58%) patients received doses ranging from 400 to 800 mg. Moreover, 76% of the patients received only one dose, whereas 22%, 26% or 24% of the critically-ill, severely-ill and moderately-ill patients received an additional dose when they did not present a reduction of their CRP levels within 48 h. 26.6 ± 7% of Tcz patients, and 18 ± 3.4% of ST patients received additional anti-inflammatory treatments for previous conditions.

### Modulation of clinical parameters

**Figure 2:**
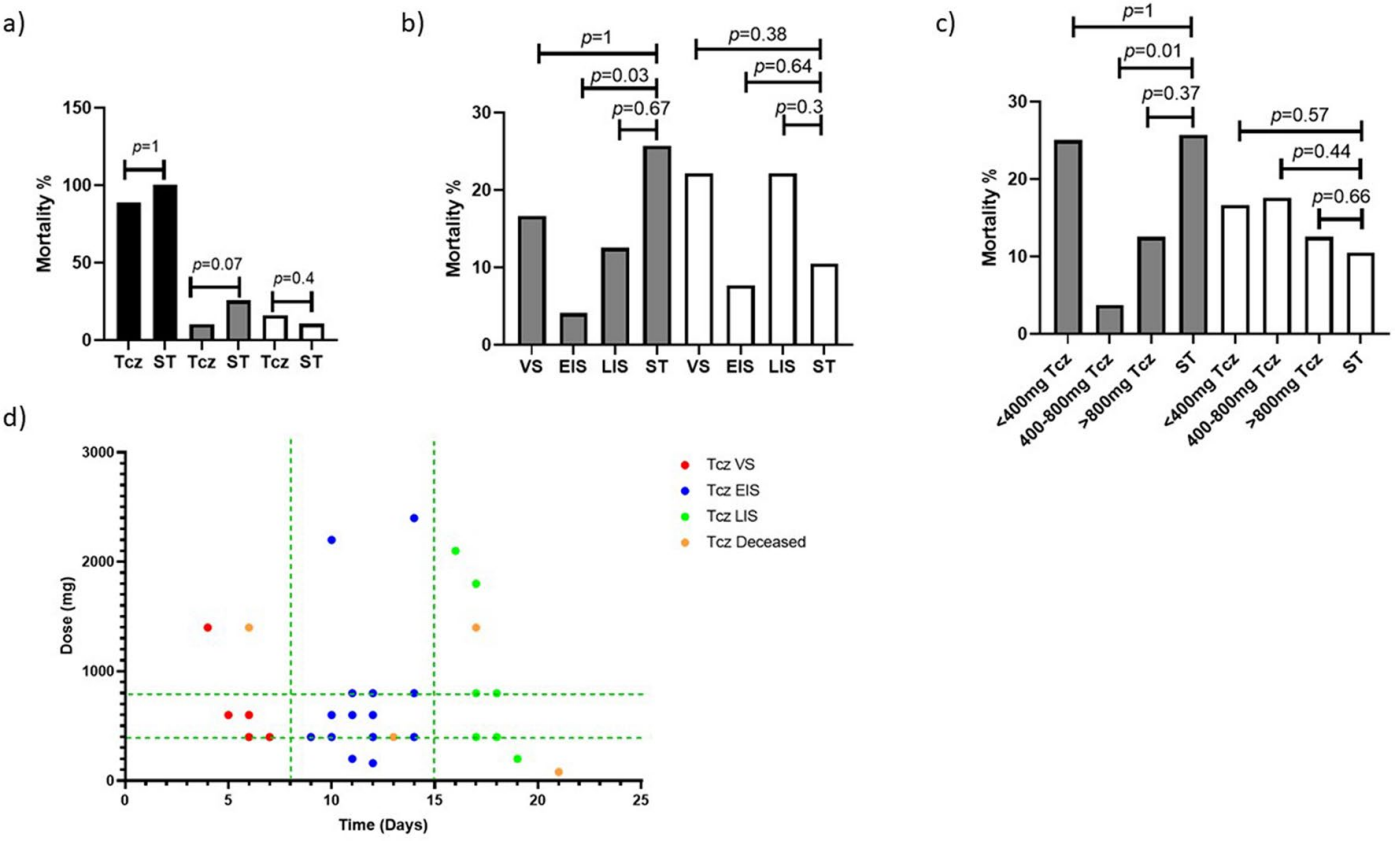
Modulation of mortality by Tocilizumab. Mortality was measured for whole groups of critically (black bars), severely (grey bars) and moderately-ill (white bars) and compared with controls having a similar disease severity (**a**), but severely and moderately-ill patients were further stratified by administration of the drug within different disease stages (**b**) and drug doses used (**c**). Finally, a qualitative multivariate analysis was performed by plotting the severely-ill surviving patients by disease stage and dose used (red, blue and green dots), as well as the severely-ill diseased patients (orange dots) (**d**). Mortality was expressed as percentage and significant differences were measured by a Fisher’s exact test, and considered as such when *p* ≤ 0.05. *Tcz* Tocilizumab; *ST* standard treatment; *VS* viral stage; *EIS* early inflammatory stage; *LIS* late inflammatory stage.

No significant difference in the mortality among the ST or Tcz groups was observed in either the critically, severely or moderately-ill patients when whole groups were compared with patients with a similar degree of severity, but receiving the ST (Fig. 2a). Nonetheless, when the Tcz group was further stratified into three categories according to the stage of COVID-19 where the drug was administered (VS, EIS and LIS), we observed a significant reduction of mortality for the severely-ill patients at the EIS (4.1% vs 25.7%; *p* = 0.03; *n* = 24). Moreover, when Tcz patients were stratified according to the dose that they received, the mortality was significantly lower (3.7% vs 25.7%; *p* = 0.01; n = 27) in the patients that received 400–800 mg than in the ST patients with a comparable disease severity (Fig. 2b,c). Such phenomenon, however, was not observed in the severely-ill patients that were treated at the VS (*n* = 6) or LIS (*n* = 9); nor with < 400 mg or > 800 mg of Tcz.

Moreover, the moderately-ill patients (*n* = 31) treated at the VS (*n* = 9), EIS (*n* = 13) or LIS (*n* = 9), with any dose, were also found to be refractory to such treatment (Fig. 2b,c). We lacked sufficient data to perform the same analysis with the critically-ill patients as only 14 were included in the study, and therefore were excluded from further analysis.

Moreover, a multivariate analysis suggests that the convergence between the aforementioned standard dose range and periods for Tcz administration represents a therapeutic strategy with enhanced probability for survival (Fig. 2d).

**Figure 3:**
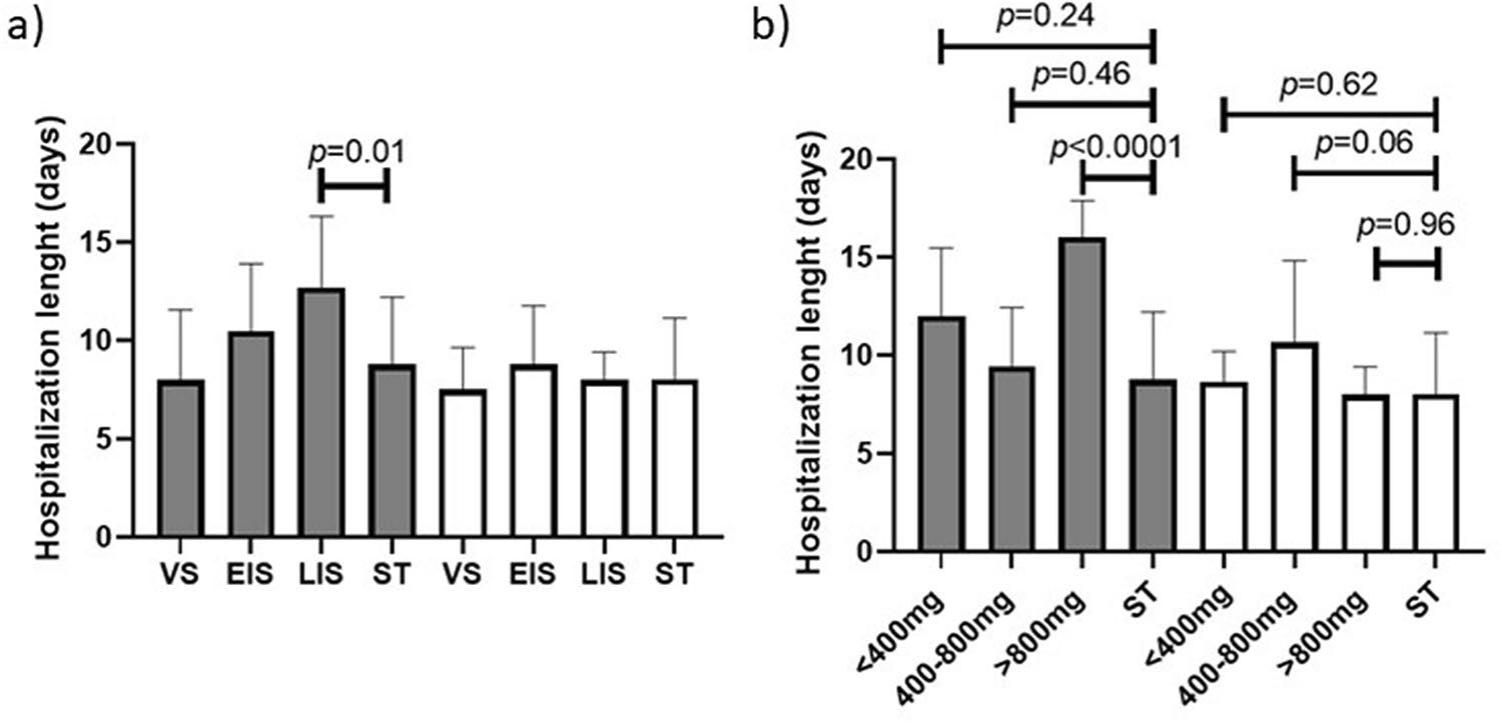
Measurement of the hospitalization length. The length of hospitalization was measured and averaged for each subgroup of disease stage when the drug was administered (**a**) and by total dose received (**b**) only for surviving patients. The gray bars represent the severely-ill patients whereas the white bars represent the moderately-ill patients. *ST* standard treatment, *Tcz* tocilizumab, *VS* viral stage, *EIS* early inflammatory stage, *LIS* late inflammatory stage. Length of hospitalization is expressed as a mean and significant differences was measured by a Student’s t-test, and considered as such when *p* ≤ 0.05.

Furthermore, Tcz was found to significantly lower the RSR 5 days after its administration for the severely (OR 2.71, CI 1.37–4.85 at 95%) and moderately-ill patients (OR 2.82, CI 1.47–4.91 at 95%). Nonetheless, 9 patients from the Tcz group and 5 from the ST group deteriorated to a critical condition, as they required MV, and Tcz was found to be unable to prevent such deterioration (OR 2.02, CI 0.51–8.0 at 95%) (Table 1). On the other hand, the drug was also unable to reduce the hospitalization length, and the severely-ill patients treated at LIS (Fig. 3a) or with > 800 mg of Tcz (Fig. 3b) seemed to have a prolonged hospitalization.

**Table 1:** Respiratory support requirements.

Parameter	Odds ratio at 95%
Reduction of RSR (severely-ill)	2.71 (1.37–4.85)
Reduction of RSR (moderately-ill)	2.82 (1.47–4.91)
Prevention of progression to MV^‡^	2.02 (0.51–8.0)

*CI* confidence interval, *RSR* respiratory support requirements, *MV* mechanical ventilation.

### Modulation of hematic and biochemical parameters

The odds of achieving normal values of CRP, LDH, ferritin and d-dimer 5 days after Tcz administration were measured, finding that the drug efficiently reduced (OR 4.82, CI 1.4–15.8) the values of the first marker, but did not produce a significant reduction of the last three in severely-ill patients (Table 2). No significant effect over these markers was observed in moderately-ill patients.

**Table 2:** Laboratory findings.

	Severe (OR at 95%CI)	Moderate (OR at 95%CI)
CRP	4.82 (1.4–15.8)	2.75 (0.6–12)
LDH	0.86 (0.19–3.9)	1 (0.05–17.9)
Ferritin	0.13 (0.01–1.3)	0.7 (0.1–5.2)
d-dimer	1.69 (0.55–5.1)	0.65 (0.13–3.18)
Total leukocytes	0.63 (0.16–2.44)	3.09 (0.69–13.7)
Lymphocytes	1.1 (0.39–3.42)	0.42 (0.09–1.85)
Total neutrophils	2.88 (1.23–8.69)	1.5 (0.35–6.6)

*CRP* C-reactive protein, *LDH* lactate dehydrogenase, *OR* odds ratio, *CI* confidence interval.

Moreover, the values of total leukocytes, lymphocytes and total neutrophils received the same treatment to establish whether Tcz has the ability to alter such parameters. We found that the drug could only significantly reduce neutrophil levels (OR 2.88, CI 1.23–8.69) in severely-ill patients (Table 2), but no other parameter was altered in such group. Also, these markers were unaltered in moderately-ill patients.

## Discussion

Overall, we observed that Tcz exerted a potent regulation of the disease when it was used to combat severe COVID-19, as CRP, neutrophil values and RSR were significantly regulated in severely-ill patients, but such effects were not observed in moderately-ill patients (except for a reduction of the RSR). However, the drug was unable to significantly reduce mortality except when it was administered to severely-ill patients at the EIS, and/or in a standard dose of 400–800 mg, pinpointing at the fact that its efficacy may be dose and timing-dependent.

The impact of Tcz on CRP and neutrophil levels was to be expected as this drug blocks the IL-6-CRP axis, a central piece of acute and chronic inflammation that has incidence on the expression of acute phase proteins, hematopoiesis, response to microbial insults and debris clearance on the setting of tissue injury^31^. Such reduction of inflammation may be in line with the observed improvement of respiratory function^32,33^, and thus with the reduced mortality for Tcz-treated patients that we found.

Tcz has shown mixed results in regards to its efficacy in the regulation of COVID-19 that range from complete inefficacy^13–16^ to a significant regulation of the disease^18–22^, and such variations may be explained by the fact that all of these studies vary in disease’s severity of the participants, total doses used and timing for their administration^34^. In fact, there is much debate surrounding Tcz’s efficacy for the treatment of COVID-19, as many details about its use are lacking, and even an individualized approach to its use has been suggested^35,36^. As a consequence, we studied the effect of this drug in a range of disease’s presentations, doses and stages, aiming at clear data about the best uses for this drug in COVID-19.

We think that the best results were found in severely-ill patients as they have an enhanced inflammatory state in comparison with moderately-ill patients, and that EIS was the best stage for Tcz administration given that immunopathology starts to develop at such moment. On the other hand, we think that Tcz’s limited ability to regulate COVID-19 at an earlier stage may be derived from the scarce participation of inflammation in the whole pathology at the VS; and that its restricted activity at later stages may occur because the inflammatory damage is already done at LIS^37^.

Additionally, the enhanced effect that a moderate dose (400–800 mg) showed on the regulation of COVID-19-derived mortality, may be explained by the fact that while a lesser dose may lack robustness, an enhanced one may propitiate secondary infections, as other researchers have found^38^.

We did not observe a reduction of hospitalization length in any scenario, nor any potential for this drug to reduce the probability of patients progressing to mechanical ventilation. But Tcz’s effects over critically-ill patients could not be correctly assessed as we found few clinical files of patients with such condition. However, the administration of Tcz at the LIS and/or in doses of > 800 mg seemed to prolong hospitalization, which may be in line with data describing an enhanced propensity for Tcz-treated patients to develop the aforementioned nosocomial superinfections^23^, and thus to present a prolonged stay at intensive care units^25^. We lack bacterial culture data from our patients as a means to confirm this hypothesis, but it seems plausible and we think that further investigation on the prophylactic use of antibiotics to avoid prolonged hospitalization in Tcz-treated COVID-19 patients would help to clarify such point.

Although all collected data was verified by another blind researcher to ensure precision, we think that our study possesses some limitations. For instance, stratifying patients by disease-severity, stage and dose used requires an enhanced power, as the subgroups formed by such strategy tend to have a reduced size. This fact was especially evident with critically-ill patients, a group that had an *n* = 14. Thus, our analysis of the potential for Tcz in the regulation of critical COVID-19 could not be completed. On the other hand, another potential limitation is the retrospective nature of our study, as no enhanced control over several variables can be exerted in such study design. Also, another concern may be that other factors, such as different strains and patient’s characteristics, may play important roles in the pathophysiology of the disease, and therefore with the efficacy of treatments^26^, in such a way that cytokine storm may not be the only, or even main factor^27^, accounting for disease outcome. In such scenario, further multivariate studies with enhanced power may be necessary to warrant more clarity in this topic.

Together, these observations suggest that COVID-19 may have a wide clinical spectrum that is not well understood, and that the efficacy of Tcz may vary according to the different clinical presentations, and especially, to the different COVID-19 stages. More research is needed to fully understand such phenomenon in order to adequate the pharmacotherapy to the different clinical presentations.

## Conclusions

Tcz has a considerable efficacy to reduce mortality and RSR, as well as CRP and neutrophil levels in severely-ill patients when used at the EIS, in doses ranging from 400 to 800 mg.

## Supplementary Information

Supplementary Table S1.

## Data Availability

Data is available upon request to the corresponding author.
