# Supplementary material for: Tocilizumab reduces COVID-19 mortality and pathology in a dose and timing-dependent fashion: a multi-centric study
Source: Sci Rep. 2021 Oct 5;11:19728. doi: 10.1038/s41598-021-99291-z (PMC8492686; doi:10.1038/s41598-021-99291-z)
Supplement: Supplementary file 1 — Supplementary Table S1. [file 41598_2021_99291_MOESM1_ESM.pdf]

Tocilizumab reduces COVID-19 mortality and pathology in a dose and timing-dependent fashion: a multi-centric study.

Alejandro Durán-Méndez, Alma Delia Aguilar-Arroyo, Emiliano Vivanco-Gómez, Eduardo Nieto-Ortega, Daniela Pérez-Ortega, Cristian Jiménez-Pérez, Karla Y. Hernández-Skewes, Guillermo Montiel-Bravo, Oscar J. Roque-Reyes, Fernanda Romero-Lechuga, Diana Medina-Santos, Perla Oriana-Román, Jorge Rafael Flores-Hernández, Juan Daniel Méndez-Coca, Daniela Montaño-Olmos, Karla Cecilia Farfán-Lazos, Miranda Tobón-Cubillos, América Viveros-Hernández, Fernando Sevilla-Castillo, Ángel Raúl Hernández-Romero, Shannat Ortega-Rodríguez, Aldo Christiaan Jardínez-Vera, María Antonieta Solís-González, Antonio Ramos de la Medina, Laura Martínez Pérez-Maldonado, Elizabeth Lagunes-Lara, Miguel Cova-Bonilla, Alberto N. Peón

Supplementary Table S1. Dosing strategy.

	<400 mg of Tocilizumab	*400-800 mg of Tocilizumab*	*>800 mg of Tocilizumab*	*Average dose*	*Patients receiving adittional doses*
Critically-ill	2 (22.2%)	2 (22.3%)	5 (55.5%)	790 mg	22%
Severely-ill	5 (12.8%)	24 (61.5%)	10 (25.6%)	790 mg	26%
Moderately-ill	8 (25.8%)	18 (58%)	5 (16.12%)	550 mg	24%
